# The Evolving Strategy of Californium-252 Neutron Intracavitary Brachytherapy in Treating Patients With Low-Lying T2 or T3 Rectal Adenocarcinoma: From Fixed to Individualized Regime With Intrarectal Peritumoral Injection of Amifostine

**DOI:** 10.3389/fonc.2021.758698

**Published:** 2021-11-18

**Authors:** Yanli Xiong, Li Shao, Jia Liu, Qian Zhou, Chongyi Li, Maojun Liao, Lei Zhang, Xiaoyan Dai, Mengxia Li, Xin Lei

**Affiliations:** ^1^ Cancer Center, Daping Hospital, Army Medical University, Chongqing, China; ^2^ Zhong Ke Pu Rui (ZKPR) Neutron Brachytherapy Center, Daping Hospital, Army Medical University, Chongqing, China

**Keywords:** rectal adenocarcinoma, radiotherapy, ^252^Cf neutron, brachytherapy, sphincter preservation

## Abstract

**Purpose:**

To retrospectively and comparatively evaluate the improvement of the efficacy and safety on the addition of ^252^Cf neutron intracavitary brachytherapy (ICBT), individualized or individualized with intrarectal peritumoral injection of amifostine (IPIA) to external-beam radiotherapy (EBRT) or concurrent chemo-EBRT in 314 patients with T2N0-1 or T3N0-1 low-lying rectal adenocarcinoma.

**Methods:**

Phase I: from 2009 to 2011, 157 patients were treated with additional ^252^Cf neutron ICBT for four fixed fractions with a total dose of 40–45 Gy-eq during the EBRT. Phase II: from 2011 to 2013, 75 patients were treated with individualized neutron ICBT delivered for two to five fractions with a total dose of 26–45 Gy-eq according to the response of tumor after concurrent chemo-EBRT. Phase III: from 2013 to 2014, 82 patients were treated with individualized ICBT protected by pretreatment IPIA.

**Results:**

The 4-year local control rates for the entire T2 and T3 patients were 69.4, 72.0, and 79.3%, while the 4-year overall survival rates were 63.1, 54.7, and 72.0% (P=0.08), and the 4-year disease-free survival rates were 55.4, 52.0, and 69.5% (P=0.053) in Phases I, II, and III, respectively. The late complication (LAC, ≥G2) rates were 33.8, 26.7, and 15.9%, respectively (P=0.012), and the serious LAC (≥G3) rates were 4.5, 4.2, and 0%, respectively, in Phases I, II, and III.

**Conclusion:**

Concurrent chemo-EBRT combined with individualized ^252^Cf neutron ICBT protected by IPIA shows promising efficacy and safety in treating low-lying T2 and T3 rectal adenocarcinoma patients without surgery opportunity or willing.

## Introduction

Low-lying rectal cancer (lower limit of tumor <6 cm from the anal verge) accounts for approximately 1/3 in rectal cancer patients who are constantly facing the difficult choice between radical resection using total mesorectal excision (TME) with poorer quality of life with stoma and sphincter preservation surgery with higher risk of disease recurrence. Although surgery has remained the cornerstone of curative treatment in rectal cancer, it is associated with postoperative mortality rates of 2 to 8%, especially for those aged over 85 years (1). Radiotherapy is one of the most important treatment in the (neo)adjuvant and first-line setting for local and local-regional rectal cancer. The achievement of pathological complete regression (pCR) occurs in 10 to 38% of local advanced rectal cancer (LARC) patients who undergo neoadjuvant chemoradiotherapy (CRT) and is associated with favorable disease-free survival (DFS) (2). However, a substantial proportion of patients is considered to be intermediate sensitive or resistant to conventional radiotherapy as about 30–40% of patients failed to achieve major regression after preoperational radiotherapy, even when concurrent chemotherapy was given as radiosensitizer (3, 4). Thus, the efforts of novel radiotherapy techniques application in rectal cancer had been made to improve the initial response to chemoradiotherapy.

For patients who refuse or are unable to receive surgery, intracavity contact X-ray or γ-ray brachytherapy is currently delivered in clinic in addition to conventional external beam radiotherapy (EBRT). Although contact X-ray radiotherapy was effective for T1 and early T2 stage low-lying rectal cancer, the treatment of T3 tumors with further contact X-ray or γ-ray (using off-axis applicator) brachytherapy must be restricted to highly selected patients, depending on the assessment of response to EBRT or concurrent chemoradiotherapy. If tumor has regressed over 80%, contact X-ray or γ-ray brachytherapy can be applied to increase the local control. If tumor has regressed less than 80%, immediate salvage surgery should be recommended to the patients (5).

However, in China, the majority of rectal patients are in advanced stage when diagnosed. For tumors initially staged as T3, the risk of lymph node spread is high (~30%) (6), which are not recommended for treating with conventional radiotherapy alone (7). Californium-252 (^252^Cf) is a source of mixed neutron/gamma rays, which has high linear energy transfer (LET) properties with special biological effects that potentially overcome the resistance of rectal adenocarcinoma cells to conventional photon rays (8). Nowadays, ^252^Cf has been used as a source of brachytherapy in treating patients with cervical, esophageal, and some other types of cancer. Our previous study also suggested a promising result of ^252^Cf neutron ICBT alone in rectal cancer patients with T1N0 rectal cancer (9). To our knowledge, the efficacy and safety profile of ^252^Cf neutron ICBT in treating T2N0-1 or T3N0-1 rectal cancer patients have not been documented. Therefore, it is of particular interest to investigate the possibility to apply ^252^Cf ICBT combining with radiotherapy or neoadjuvant chemoradiotherapy in this clinical commonly encountered cohort of rectal cancer. In the current study, we reported the improvement of safety and efficacy data on the addition of ^252^Cf neutron ICBT (phase I: from January of 2009 to November of 2011), individual (phase II: from November of 2011 to January of 2013), or individual one protected by intrarectal peritumoral injection of amifostine (phase III: from January of 2013 to August of 2014) to external-beam radiotherapy (EBRT) or concurrent chemo-EBRT in 317 patients with T2N0-1 or T3N0-1 low-lying rectal adenocarcinoma.

## Materials and Methods

### Patients

**Figure 1:**
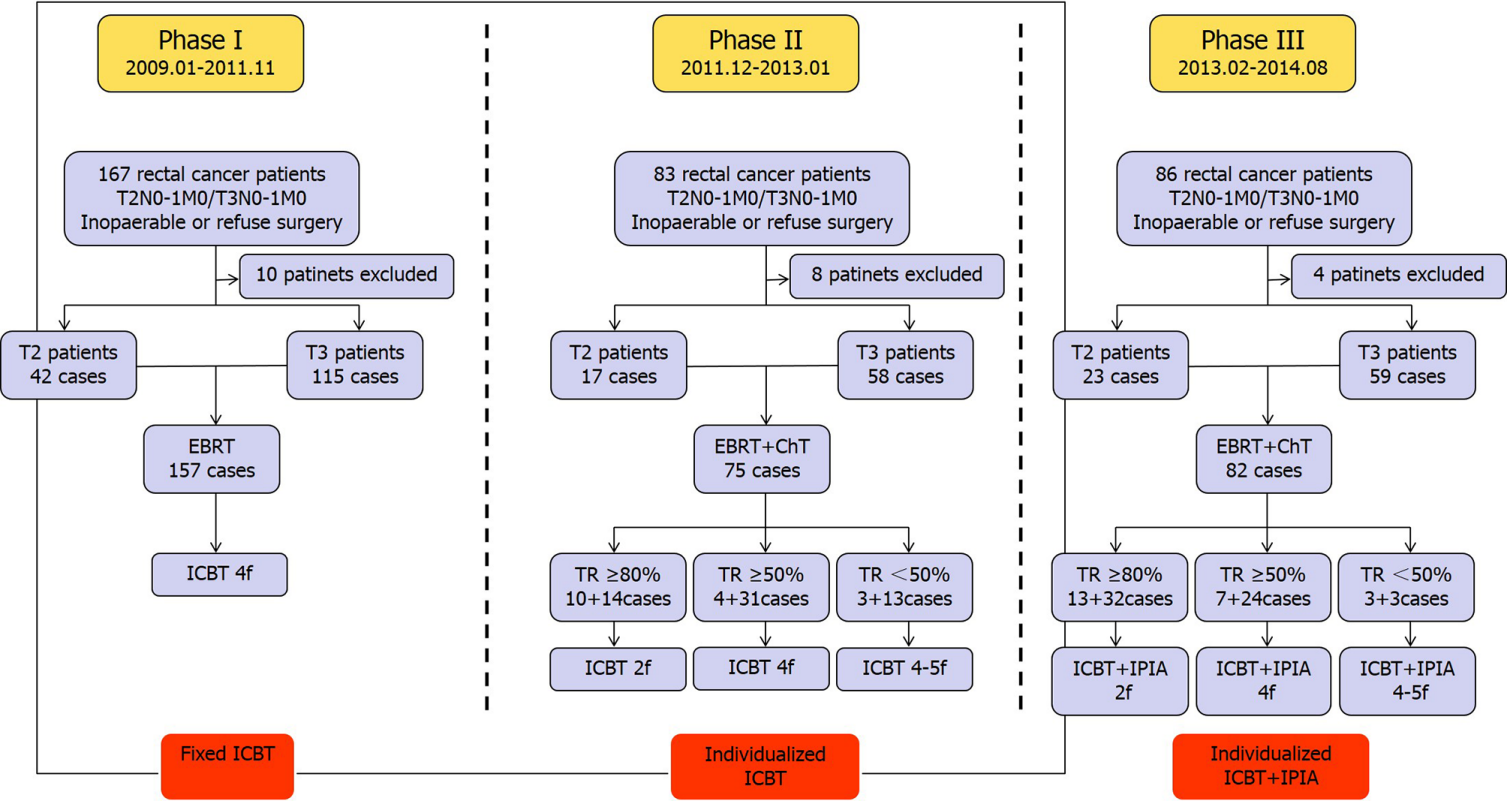
Diagram for the three phases of current study. EBRT, external beam radiotherapy; ICBT, neutron intracavitary brachytherapy; ChT, chemotherapy; TR, tumor regression; IPIA, intrarectal peritumoral injection of amifostine; f, fraction.

A total of 317 patients with low-lying rectal adenocarcinoma (<6 cm from the anal verge) staged with T2N0-1M0 or T3N0-1M0 who were inoperable and firmly refused surgery procedures were treated using ^252^Cf neutron ICBT at our center (157 patients in Phase I, 75 patients in Phase II, and 82 patients in Phase III). All histopathological diagnoses were confirmed in colonoscopy biopsy tissue in Pathology Department of Daping Hospital. Clinical staging processes were guided by institutional protocol and determined according to the Union for International Cancer Control (UJCC) TNM 7^Th^ edition. In general, patients underwent pelvic magnetic resonance imaging (MRI), colonoscopic endoanal ultrasound scanning (EUS), and chest and total abdominal computed tomography (CT). Alternatively, positron emission tomography CT (PET-CT) was ordered instead of chest and total abdominal CT to exclude distal metastasis. The eligibility of surgery and peri-operational treatment was determined by institutional colorectal cancer multidisciplinary team (MDT), including medical oncologists, gastrointestinal surgeons, radiation oncologists, pathologists, and radiologists. Patients with full understanding of their disease and all the treatment options who were inoperable or refused to receive surgery and strongly preferred ^252^Cf radiotherapy were enrolled in our center for ^252^Cf ICBT combined EBRT with or without chemotherapy, after signed a consent form. The pretreatment screens including Karnofsky scoring, whole blood count, serum carcinoembryonic antigen (CEA), and biochemistry were then conducted for all patients enrolled, and only those who met general criteria for radiotherapy and chemotherapy then received scheduled therapy, which will be described later in this section. Retrospective medical record review was performed for all patients, and only eight patients who failed to complete follow-up visit were excluded in this analysis. Flow diagram of this study is shown in **Figure 1**, and the baseline of patient characteristics is summarized in **Table 1**.

**Table 1:** The characteristics of rectal cancer patients with T2N1M0 or T3N1M0 in phases I–III.

T Stage	T2(n)			P	T3(n)			P
Phase	I	II	III		I	II	III	
**Age (Mean, years)**	62.88 ± 12.75	61.41 ± 15.42	58.04 ± 12.97	0.332	61.57 ± 13.86	61.14 ± 11.52	59.71 ± 14.54	0.171
<70	30	11	20		81	45	45	
≥70	12	6	3		34	13	14	
**Gender**								
Male	28	10	14	0.815	69	34	42	0.275
Female	14	7	9	46	24	17		
**Involvement (circumstance)**								
1/3	24	11	11	0.492	69	19	13	**0.000**
1/2	15	6	12	23	21	30		
2/3	3	0	0	23	18	16		
**Lymph node statue**								
Negative	38	13	17	0.173	82	34	42	0.202
Positive	4	4	6	33	24	17		
**Distance from anal verge**								
<3	11	5	14	**0.020**	25	10	8	0.412
≥3	31	12	9	90	48	51		
**Tumor size (Mean, cm)**	3.71 ± 1.15	3.29 ± 0.83	3.61 ± 0.78	0.348	3.83 ± 1.26	4.41 ± 1.35	4.50 ± 1.07	**0.001**
≤3 cm	24	12	11		69	20	13	
>3 cm	18	5	12		46	38	46	
**Total number**	42	17	23		115	58	59	

LC = Local contral rate, DFS = Disease-free survivl rate, OS = Overal survival rate.Bold means that the P value is less than 0.05.

### External Beam Radiotherapy

#### Conventional External Beam Radiotherapy (Phase I)

Four small fields (8 cm × 10 cm) EBRT with a total dose of 39.6 Gy (0.55 Gy/field, 2.2 Gy/f/d, 18f) were administered for T_2_N_0-1_M_0_ patients. On the other hand, three whole pelvic fields (laterals with wedge) EBRT with a total dose of 42 Gy (2.1 Gy/f/d, 20f) were administered for T_3_N_1_M_0_ patients. While, T_3_N_0_M_0_ patients were delivered using whole pelvic field with a total dose of 21 Gy (2.1 Gy/f/d, 10f) plus small field with a total dose of 19.8 Gy (2.2 Gy/f/d, 9f).

#### 3-D Conformal EBRT (Phase II)

In T2 cohort, T2N0-1M0 patients were irradiated using regional perirectal 3-D conformal radiotherapy with a total dose of 39.6 Gy (2.2 Gy/f/d×18f). In T3 cohort, T3N1M0 patients received whole pelvic 3-D conformal radiotherapy with a total dose of 42 Gy (2.1 Gy/f/d, 20f). T3N0M0 patients received whole pelvic 3-D conformal radiotherapy with a total dose of 21 Gy (2.1 Gy/f/d) plus regional perirectal 3-D conformal radiotherapy with a total dose of 19.8 Gy (2.2 Gy/f/d×9f) radiotherapy.

#### Intensity-Modulated Radiation Therapy (Phase III)

In T2 cohort, T2N0M0 patients were irradiated using regional rectal tumor IMRT with a total dose of 39.6 Gy (2.2 Gy/f/d, 18f). While T2N1M0 patients received regional rectal tumor and perirectal lymph node IMRT with a total dose of 44 Gy (2.2 Gy/f/d, 20f).

In the T3 cohort, T3N0M0 patients received whole pelvic IMRT with a total dose of 40 Gy (GTV-1, 2.0 Gy/f/d, 20f) and regional rectal tumor IMRT with a total dose of 44 Gy (GTV-2, 2.2 Gy/f/d, 20f).

While T3N1M0 patients received whole pelvic IMRT with a total dose of 42 Gy (GTV-1, 2.0 Gy/f/d, 21f) or/and internal iliac lymph node IMRT with a total dose of 46.2 Gy (GTV-3, 2.4 Gy/f/d, 21f), and regional rectal tumor and perirectal lymph node IMRT with a total dose of 46.2 Gy (GTV-2, 2.2 Gy/f/d, 21f).

#### Concurrent Chemotherapy (Phases II and III)

In general, all 5-fluorouracil-based chemotherapy regimens on day 1 of EBRT and last at least 3 days were accepted in this study. Typical chemotherapy regimens used in the current study include (a) 5-FU: bolus 5-FU 0.5 g/m2 days 1–2 followed by infusional 5-Fu 1 g for 48 h every 14 days for two cycles; (b) capecitabine 2,500 mg/m2 daily days 1–14 every 21 days for two cycles; (c) S-1: 50 mg twice daily days 1–21 every 28 days for two cycles.

### Evaluation of Tumor Response

Tumor response was evaluated at 2–3 weeks post completion of EBRT by the same clinical and radiologic tools used in the baseline assessment of tumor extent. All patients considered to be complete clinical responders according to stringent criteria of clinical, endoscopic, and radiologic findings were treated without immediate radical surgery, as described elsewhere (10). Briefly, the criteria for considering cCR were the absence of residual ulceration, mass, or mucosal irregularity at clinical/endoscopic assessment. Whitening of the mucosa and the presence of neovasculature (teleangiectasia) were accepted features of cCR. In cases of the presence of clinical or endoscopic features of incomplete response to initial CRT or the radiologic evidence of residual disease within the mesorectum were diagnostic of incomplete clinical response, ^252^Cf ICBT was recommended. The percentages of tumor regression were assessed again by digital rectal examination and anoscope at 4–6 weeks after the completion of ICBT.

### ^252^Cf neutron Radiotherapy

ZunRui LZH-1000 ^252^Cf neutron brachytherapy devices (ZunRui company, Shenzhen, China) were used. The large-size neutron source (length: 10 mm, diameter: 5 mm) was used in this study. The neutron source activity was 520–140 μg. The three or four channels, 3 or 3.5 cm in diameter off-axis applicators were selected in terms of the involvement of tumor’s anatomical, physiological conditions of patients during ^252^Cf neutron ICBT.

#### ^252^Cf neutron ICBT (Phase I)

The fixed four fractions were implemented with a total neutron brachytherapy dose of 40–45 Gy (11–14 Gy/f/w) at the dose reference point defined on the anal canal mucosal surface during the EBRT. The dose calculation method and detailed operation procedures of ^252^Cf neutron ICBT were described in our previous study (9).

#### Individual ^252^Cf Neutron ICBT (Phase II)

**Figure 2:**
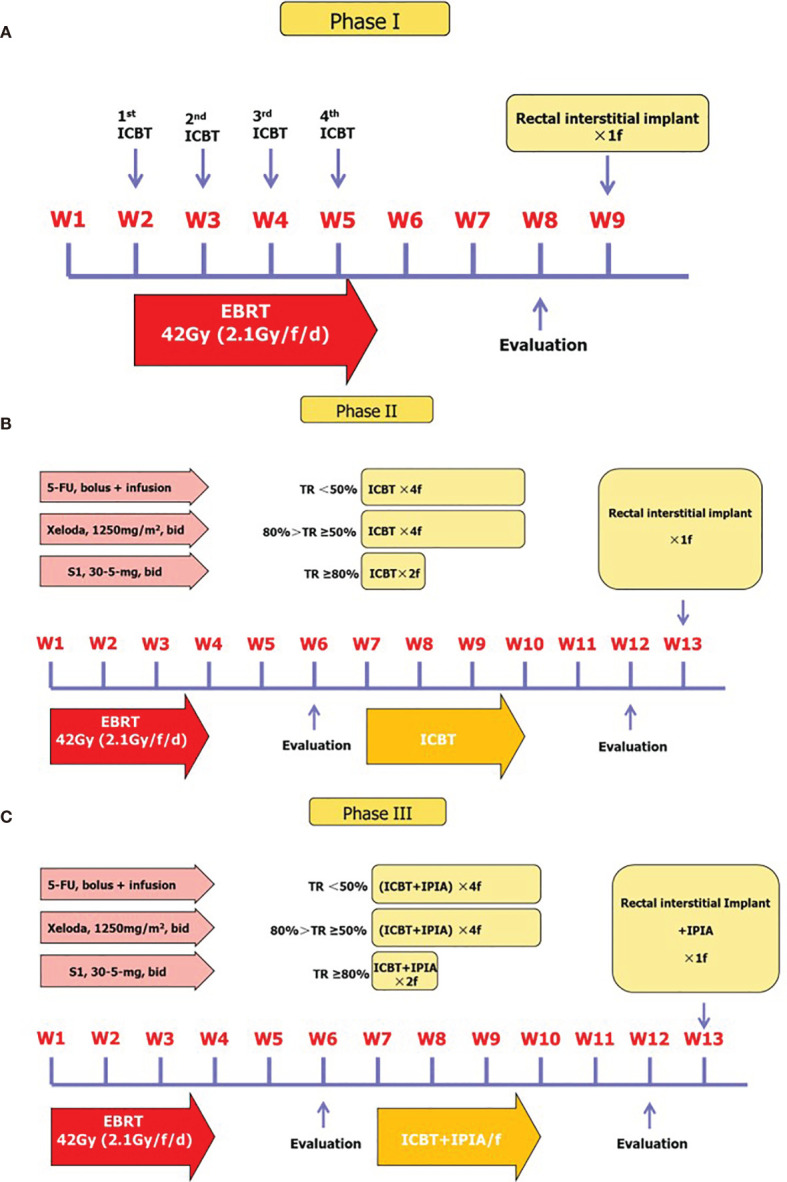
Treatment protocols for each phase of the current study. **(A)** Phase I treatment protocol. **(B)** Phase II treatment protocol and algorithm for patients with different response to initial therapy. **(C)** Phase III treatment protocol.

Two weeks after administration of chemo-EBRT, the fractions of individualized ^252^Cf neutron ICBT, depending on the assessment of response after the external beam chemoradiotherapy, were administered once a week. If the tumor has regressed above 80%, two fractions of ^252^Cf neutron ICBT were applied once a week. If the tumor has regressed above 50–80%, four fractions of ^252^Cf neutron ICBT were applied. If the tumor has regressed less than 50% after concurrent chemoradiotherapy, another one fraction of rectal interstitial implant ^252^ Cf brachytherapy with neutron brachytherapy dose of 13–15 Gy was delivered to three patients with residual lesions after completion of the abovementioned radiotherapy (**Figure 2**).

#### Individual ^252^Cf Neutron ICBT Protected by IPIA (Phase III)

The individualized ^252^Cf neutron ICBT was delivered for flexible two to four fractions with total neutron brachytherapy dose of 26–45 Gy in 11–13Gy/f/w with or without another one fraction of rectal interstitial implant in 82 patients according to the response of tumor after concurrent chemo-EBRT. In addition, the patients were injected amifostine 10 ml into mucosa and submucosa peritumorally (amifostine 0.4g + 9.5 ml 0.9% NaCl diluted) around the base of lump in the anus by local anesthesia at 30 min before the ICBT from first to fourth fraction (**Figure 1**).

#### ^252^Cf Neutron Rectal Interstitial Implant

One fraction of rectal interstitial implant was given to patients with residue tumors at up to 4 weeks post-ICBT/EBRT or ICBT/CCRT combinational therapy. The small-size neutron source was used: the length was 5 mm, the diameter was 2 mm, and its activity was 250–46 μg. The interstitial implant was given by an average three or four needles (diameter=3 mm) with spacing of 0.5–1 cm between the needles with the patient in the knee chest position with anal local anesthesia (9). Eight T2 and 14 T3 patients with residue tumor were given a fraction of rectal interstitial implant (neutron brachytherapy dose 11–13 Gy/f).

### Follow-Up

All patients completed the treatment and were assessed every 3 months in the first 2 years after completion of treatment and every 6 months thereafter. Treatment-related complications were categorized according to the Common Terminology Criteria for Adverse Events version of the Radiation Therapy Oncology Group (RTOG).

### Statistical Analysis

SPSS 17.0 software was used for statistical analyses. Kaplan-Meier method was performed to calculate and compare the 5-year OS and FFS rate in rectum. Discrete data are shown as frequencies (percentages); continuous data are shown as means ± SE.

## Results

### Patients

A total 82 patients with T2 tumor and 232 patients with T3 tumor were enrolled in this study according to the inclusion and exclusion criteria described in *Methods* section. In T2 cohort, 14 patients were with positive pelvic lymph node and 68 with negative pelvic lymph node. The medium age of this cohort was 61.3 years, and 52 patients were male and 30 were female. The ^252^Cf neutron therapy is a brachytherapy. The area of tumor lesion is considered to be closely related to the efficacy and adverse effects; therefore, we measured the area of tumor invasion under direct vision of colonoscopy. The proportion of patients with distance from anal verge less than 3 cm was higher in phase III (P=0.020), and 35 patients with a long diameter of tumor lesion (Tumor size) more than 3 cm. In the T3 cohort, 74 patients were with positive lymph node and 158 with negative lymph node. The medium age of this cohort was 61.5 years, and 145 patients were male and 87 were female. Forty-three patients with distance from anal verge less than 3 cm, and the proportion of patients with large diameter of tumor lesion more than 3 cm was higher in phase III (P=0.001). The basic information of enrolled patients in this study are displayed in **Table 1**.

### Treatment Exposure

All enrolled patients completed the required treatment regime. In the T2 cohort, 42 patients received radiotherapy alone for the initial treatment phase I, and 40 of them received concurrent chemoradiotherapy and sequential chemotherapy in the subsequent treatment phases II and III. In the T3 cohort, 115 patients received radiotherapy alone for the initial treatment phase I, and 117 of them received concurrent chemoradiotherapy and sequential chemotherapy in the subsequent treatment phases II and III. This bias was due to the shift of our standard-of-care protocols for the neoadjuvant therapy of rectal cancer in our institution in 2011.

The assessments of initial tumor response were performed at 2–3 weeks post the completion of initial treatment phase, since the interval between completion of therapy and assessment of initial response was shorter than conventional tumor response assessment of neoadjuvant therapy considering the subsequent individualized ICBT. The clinical complete regression (cCR) was rare, and we therefore categorized tumor response to three levels: tumor regression over 80% (≥80%), between 50 and 80% (<80 but ≥50%), and less than 50% (<50%) (**Figure 1**). According to our clinical experience, the population with tumor regression over 80% had very good chance to develop cCR eventually. In phases II and III, 69 patients (23 in T2 and 46 in T3) with tumor response ≥80% received one to two additional factions of ICBT, 66 patients (11 in T2 and 55 in T3) with tumor response <80 but ≥50% received three to four additional fractions of ICBT, and 22 patients (6 in T2 and 16 in T3) with tumor response <50% received additional four to five fractions of ICBT. The basic protocol of our current treatment regime in this study is shown in **Figure 2**.

### Efficacy

#### Local Control

**Figure 3:**
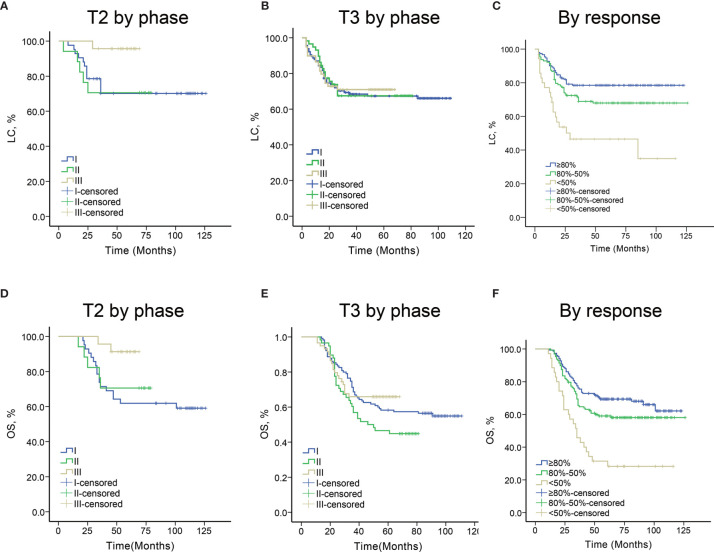
**(A)** Local control curves by different initial response groups. **(B)** Overall survival curves by different initial response groups. **(C)** Local control curves in patients with T2 tumors by different phases of the current study. **(D)** Overall survival curves in patients with T2 tumors by different phases of the current study. **(E)** Local control curves in patients with T3 tumors by different phases of the current study. **(F)** Overall survival curves in patients with T3 tumors by different phases of the current study.

Besides survival, a major problem in LARC is the threat of local recurrence, not only because of the limited therapeutic options but especially because of the poor quality of life (**Figure 3**). In the T2 cohort, the 4-year local control rate of this study is 71.4% (30/42), 76.5% (13/17), 100% (23/23) in phases I, II, and III, respectively (P=0.008). In the T3 cohort, the 4-year local control rate of this study is 68.7% (79/115), 70.7% (41/58), 71.2% (42/59) in phases I, II, and III, respectively (P=0.947) (**Table 2**). Noteworthy, 22 patients with residual lesions received rectal interstitial implant, and local control was achieved in 14 patients (14/22,63.7%). Stratified analyses suggest that in T2 cohort, the local control rate is associated with initial tumor response (Tumor regression) and individualized ICBT treatment regime, but not associated with lymph node status, age, gender, involvement, distance from the anal verge, and tumor size (**Table 3**). While in the T3 cohort, the local control rate is associated with initial tumor response (Tumor regression), lymph node status, involvement, and tumor size, but not associated with age, gender, distance from the anal verge, and individualized ICBT treatment regime. Multivariate analyses showed that tumor response (Tumor regression), lymph node status, involvement, and tumor size were independent risk factors of the local control rate in the T3 cohort (**Table 4**).

**Table 2:** The 4-year result of rectal cancer patients treated with radiotherapy for T2N1M0, T3N1M0, or both, respectively, in phases I–III.

T stage	T2 (%)			P	T3 (%)			P	T2 and T3 (%)			P
Phase	I	II	III		I	II	III		I	II	III	
**LC**	71.4	76.5	100	**0.008**	68.7	70.7	71.2	0.947	69.4	72.0	79.3	0.264
**DFS**	61.9	64.7	95.7	**0.011**	53.0	48.3	59.3	0.508	55.4	52.0	69.5	0.053
**OS**	64.3	70.6	91.3	**0.047**	62.6	50.0	64.4	0.202	63.1	54.7	72.0	0.080
**LAC (≥G2)**	28.6	23.5	13.0	0.353	35.6	27.6	16.9	**0.034**	33.8	26.7	15.9	**0.012**

LC = Local contral rate, DFS = Disease-free survivl rate, OS = Overal survival rate.Bold means that the P value is less than 0.05.

**Table 3:** Univariate and multivariate analyses of factors associated with LC, DFS, and OS for T2 rectal cancer patients, respectively.

	LC				DFS				OS			
Characteristic	Univariate Analyses		Multivariate Analyses		Univariate Analyses		Multivariate Analyses		Univariate Analyses		Multivariate Analyses	
	HR (95% CI)	P	HR (95% CI)	P	HR (95% CI)	P	HR (95% CI)	P	HR (95% CI)	P	HR (95% CI)	P
Phase (I, II, III)	0.512 (0.263–0.997)	**0.049**	0.149 (0.019–1.163)	0.069	0.179 (0.041–0.774)	**0.021**	Excluded		0.195 (0.045–0.850)	**0.030**	Excluded	
Age (<70, ≥70)	2.052 (0.809–5.203)	0.130	Excluded		1.321 (0.588–2.966)	0.500	Excluded		2.198 (0.984–4.912)	0.55	Excluded	
Gender (Male, Female)	0.619 (0.221–1.738)	0.363	Excluded		1.240 (0.570–2.701)	0.588	Excluded		0.703 (0.291–1.695)	0.432	Excluded	
Involvement (circumstance, <1/3, 1/3–2/3, ≥2/3)	1.003 (0.448–2.248)	0.993	Excluded		1.135 (0.593–2.174)	0.703	Excluded		1.131 (0.565–2.261)	0.728	Excluded	
Lymph node status (Negative, Positive)	1.026 (0.297–3.545)	0.967	Excluded		1.360 (0.512–3.613)	0.537	Excluded		1.453 (0.542–3.897)	0.458	Excluded	
Distance from anal verge (<3, ≥3)	0.869 (0.337–2.241)	0.771	Excluded		0.686 (0.313–1.506)	0.348	Excluded		0.748 (0.331–1.691)	0.485	Excluded	
Tumor size (Mean, ≤3 cm, >3 cm)	1.313 (0.521–3.309)	0.564	Excluded		1.356 (0.628–2.928)	0.437	Excluded		1.283 (0.576–2.857)	0.542	Excluded	
Tumor regression (≥80%, 50–80%, <50%)	2.135 (1.127–4.047)	**0.020**	0.797 (0.208–3.055)	0.740	0.711 (0.258–1.959)	0.509	Excluded		0.612 (0.217–1.722)	0.352	Excluded	

LC = Local contral rate, DFS = Disease-free survivl rate, OS = Overal survival rate.Bold means that the P value is less than 0.05.

**Table 4:** Univariate and multivariate analyses of factors associated with LC, DFS, and OS for T3 rectal cancer patients, respectively.

	LC				DFS				OS			
Characteristic	Univariate Analyses		Multivariate Analyses		Univariate Analyses		Multivariate Analyses		Univariate Analyses		Multivariate Analyses	
	HR (95% CI)	P	HR (95% CI)	P	HR (95% CI)	P	HR (95% CI)	P	HR (95% CI)	P	HR (95% CI)	P
Phase (I, II, III)	0.954 (0.720–1.264)	0.742	Excluded		0.930 (0.742–1.165)	0.529	Excluded		0.978 (0.772–1.239)	0.852	Excluded	
Age (<70, ≥70)	1.358 (0.841–2.193)	0.211	Excluded		1.165 (0.791–1.717)	0.439	Excluded		1.084 (0.712–1.652)	0.706	Excluded	
Gender (Male, Female)	0.911 (0.564–1.471)	0.704	Excluded		0.981 (0.673–1.430)	0.920	Excluded		1.026 (0.689–1.528)	0.900	Excluded	
Involvement (circumstance, <1/3, 1/3–2/3, ≥2/3)	1.462 (1.105–1.934)	**0.008**	Excluded		1.364 (1.092–1.704)	**0.006**	Excluded		1.290 (1.019–1.634)	**0.035**	Excluded	
Lymph node status (Negative, Positive)	2.417 (1.522–3.839)	**0.000**	2.171 (1.357–3.474)	**0.001**	2.139 (1.473–3.104)	**0.000**	1.962 (1.343–2.867)	**0.000**	2.445 (1.653–3.615)	**0.000**	2.445 (1.653–3.615)	**0.000**
Distance from anal verge (<3, ≥3)	0.713 (0.414–1.228)	0.223	Excluded		0.812 (0.518–1.273)	0.364	Excluded		0.717 (0.451–1/140)	0.159	Excluded	
Tumor size (Mean, ≤3 cm, >3 cm)	1.454 (1.098–1.924)	**0.009**	Excluded		1.333 (1.066–1.667)	**0.012**	Excluded		1.252 (0.988–1.587)	0.063	Excluded	
Tumor regression (≥80%, 50–80%, <50%)	1.753 (1.259–2.440)	**0.001**	1.594 (1.143–2.222)	**0.006**	1.550 (1.178–2.039)	**0.002**	1.417 (1.074–1.869)	**0.014**	1.598 (1.205–2.119)	**0.001**	1.449 (1.089–1.926)	**0.011**

LC = Local contral rate, DFS = Disease-free survivl rate, OS = Overal survival rate.Bold means that the P value is less than 0.05.

#### Endoscopic Changes at 12–24 Months After Completion of ICBT

**Figure 4:**
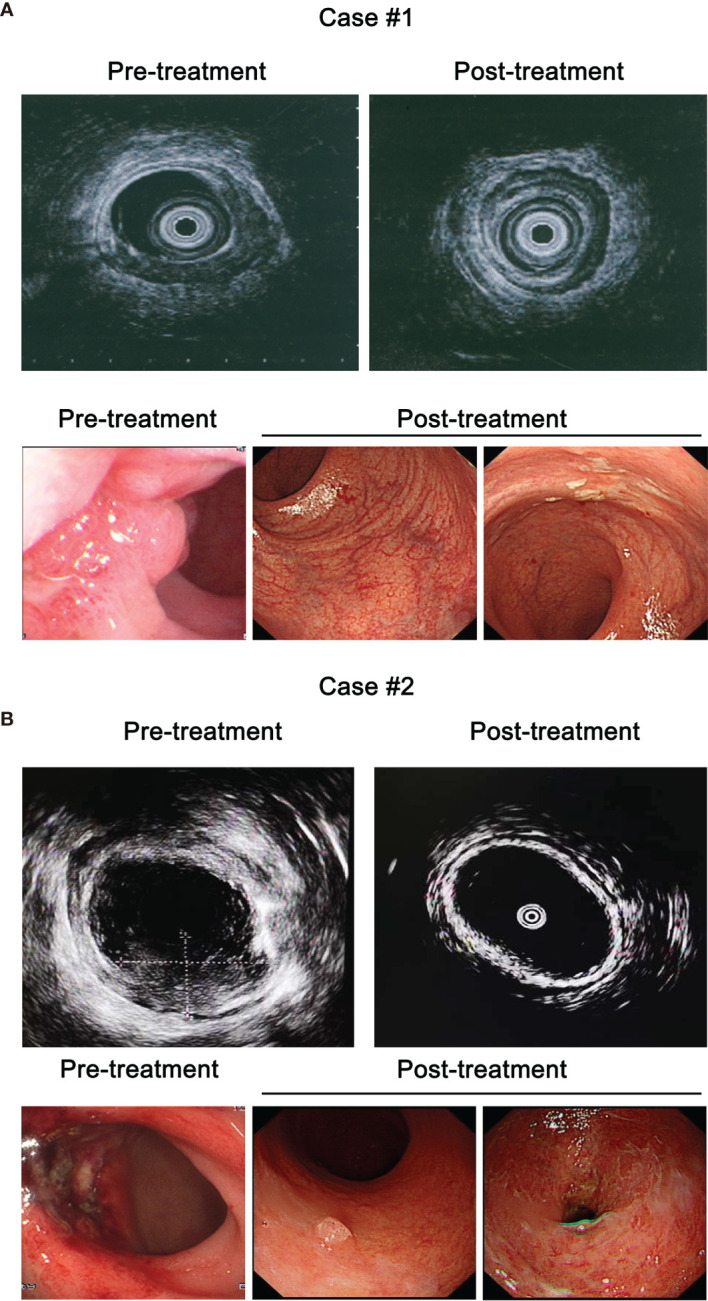
Clinical images of representative patients in the current study. **(A)** Pre- and post-treatment colonoscopic endoanal ultrasound scanning (EUS, upper panel) and colonoscopy (lower panel) images of case #1 with T2 tumor. **(B)** Pre- and post-treatment EUS (upper panel) and colonoscopy (lower panel) images of case #2 with T3 tumor.

At tumor regression assessment after completion of ICBT, we have observed significant differences in morphological changes under colonoscopy among patients with various response to initial CRT. In patients with initial response over 80%, regional teleangiectasia were commonly observed after achieving complete tumor regression. In patients with initial response between 50 and 80%, scattered whitening of the mucosa, which represents localized fibrosis, was also observed in some cases on the background of teleangiectasia. However, in patients with initial response below 50%, ulceration can be formed and covered with larger area of whitened mucosa with black spotted tissue on the surface (**Figure 4**). Our side projects have revealed the existence of cancer-initiating cells in this type of lesion.

#### Survival

In the T2 cohort, the 4-year DFS rate of this study is 61.9% (26/42), 64.7% (11/17), 95.7% (22/23) in phases I, II, and III, respectively (P=0.011) (**Table 2**). In the T3 cohort, the 4-year DFS rate of this study is 53.0% (61/115), 48.3% (28/58), 59.3% (35/59) in phases I, II, and III, respectively (P=0.508) (**Table 2**). Stratified analyses suggest that in the T2 cohort, the DFS rate is associated with individualized ICBT treatment regime, but not associated with lymph node status, age, gender, involvement, distance from the anal verge, tumor size, and initial tumor response. While in the T3 cohort, the DFS rate is associated with involvement, lymph node status, tumor size, and initial tumor response, but not associated with age, gender, distance from the anal verge, and individualized ICBT treatment regime. Multivariate analyses showed that lymph node status and initial tumor response were independent risk factors of DFS rate in the T3 cohort.

In the T2 cohort, the 4-year OS rate of this study is 64.3% (27/42), 70.6% (12/17), 91.3% (21/23) in phases I, II, and III, respectively (P=0.047) (**Table 2**). In the T3 cohort, the 4-year DFS rate of this study is 62.6% (72/115), 50.0% (29/58), 64.4% (38/59) in phases I, II, and III, respectively (P=0.202) (**Table 2**). Stratified analyses suggest that in the T2 cohort, the OS rate is associated with individualized ICBT treatment regime, but not associated with lymph node status, age, gender, involvement, distance from the anal verge, tumor size, and initial tumor response. While in the T3 cohort, the OS rate is associated with involvement, lymph node status, and initial tumor response, but not associated with tumor size, age, gender, distance from the anal verge, and individualized ICBT treatment regime. Multivariate analyses showed that lymph node status and initial tumor response were independent risk factors of DFS rate in the T3 cohort.

#### Efficacy by Initial Response

Regarding the correlation of response to CRT to local recurrence and survival in rectal patients, we evaluated the LR and survival of patients with different initial responses in our study. In the good response group (>80%), the 4-year local control rate of this study is 64.6% (53/82), 62.5% (15/24), 71.1% (32/45) in phases I, II, and III, respectively (P=0.727). In the intermediate response group (<80 and >50%), the 4-year local control rate of this study is 48.4% (30/62), 57.1% (20/35), 64.5% (20/31) in phases I, II, and III, respectively (P=0.339). Although the case number is limited, in the poor response group (<50%), the 4-year local control rate of this study is 0% (0/13), 25.0% (4/16), 83.3% (5/6) in phases I, II, and III, respectively (P=0.000).

### Toxicity

During the treatment, acute adverse effects involving the rectum tended to be mild (Grades 1 and 2), suggesting the overall tolerance of this regime was good. The incidence of late complications (≥G2) was 33.8, 26.7, and 15.9% in phases I, II, and III, respectively (P=0.012) (**Table 2**). In the T2 cohort, the incidence of late complications (≥G2) was 28.6, 23.5, and 13.0% in phases I, II, and III, respectively (P=0.353). The serious late complication (G3) rate was 2.38%, 0 and 0 in phase I, II, and III, respectively (P=1). In the T3 cohort, the incidence of late complications (≥G2) was 35.6, 27.6, and 16.9% in phases I, II, and III, respectively (P=0.353). The serious late complication (G3) rate was as follows: 3.9% (9/232) rectal bleeding occurred in 26 patients, rectal frequent urgency was reported in 54 patients, and both rectal bleeding and frequent urgency were reported in 15 patients. Nine patients with both rectal bleeding and frequent urgency developed grade 3 toxicity and finally proceeded to colostomy in phases I and II, and none developed grade 3 toxicity in phase III. Hence, the serious late complication (G3) rate was 3.9% (9/232) (**Table 3**). Among patients who developed late toxicity, those with tumor located <2 cm from the anal verge tended to show symptoms of frequent urgency, while those with tumor located >4 cm from the anal verge developed bleeding. For relief of symptoms of frequent urgency, patients were administered non-steroidal anti-inflammatory drugs per rectum. Bleeding was treated by rectal administration of liquid solution norepinephrine (2–4 mg).

## Discussion

For LARC patients without surgery opportunity or willing, radiotherapy is the most important, if not the only, local treatment. To overcome the resistance to conventional radiotherapy in rectal cancer patients, we introduced a novel radiotherapy with powerful radiation source in treating these patients in our center. For the last decade, we have been using individualized ^252^Cf neutron ICBT in combination with EBRT to treat over 300 LARC patients with T2 or T3 tumor who are inoperable or patients who refused, and 317 patients who have complete follow-up data were included in this study.

Current investigation has included three distinct but evolving strategies to apply this novel radiotherapy to patients with LARC, which represents our explorative path in improving efficacy and reducing toxicity. Before starting this effort in rectal cancer, we previously employed ICBT in cervical cancer radiotherapy, and it was well tolerated and effective. Thus, in the beginning of this study (Phase I), we basically adopted the fixed four fractions ICBT regime used during the EBRT for rectal cancer patients. The efficacy of ICBT was as expected, while the rate of adverse effects was higher due to the lower radiation tolerance of rectum mucous compared to cervical tissue. We then designed a second regime, which is much more individualized, adapted to the response of initial radiotherapy. In Phase II, we tried to give less fractions in patients who reached better response after concurrent chemoradiotherapy with the aim to reduce the adverse effects without sacrificing the efficacy. On one hand, the toxicity of this regime was reduced in digits when compared to Phase I, especially in T3 cohort. On the other hand, the PFS and OS were slightly reduced in digits than those of Phase I, despite the higher local control. The unexpected survival reduction may be due to the clinical characteristics associated with poorer prognosis of included patients, such as larger tumor volume, more tumor involvement. The interval to evaluate initial responses between EBRT and ICBT, which increased the time of whole radiotherapy regime, could be another possibility to take into account. Taken together, we concluded Phase II exploration was successful in regard to better local control and lower toxicity, although the toxicity was still unacceptable when G3 toxicity was observed in 4% of patients, which need further surgical treatment. In Phase III of the current study, we introduced a much powerful pretreatment, amifostine injection, to further reduce the toxicity of individualized ICBT. By using this pretreatment, we finally diminished the G3 toxicity as well as reduced the overall adverse effects. In addition, the local control and survival were significantly improved in digits.

For patients who received radiotherapy as definite therapy, radiotoxicities have to be taken into account (11). Our current study showed that without any radioprotective agents, especially in Phase I, radiotherapy or combinational treatment rendered 30% grade 2 or above proctitis in low-lying rectal cancer patients, and among them, 7–8% grade 3 proctitis which is in need of surgical procedure. Therefore, to avoid this dilemma, we introduced amifostine as radioprotector in our individualized ICBT in Phase III. Amifostine is conventionally administered intravenously before chemotherapy or radiotherapy. A common and potentially dangerous side effect in patients receiving intravenous amifostine is hypotension, which has been reported to occur in approximately 62% of patients treated at a dose of 910 mg/m^2^. To balance the dose of amifostine and severity of its adverse effects, a number of amifostine delivery routes have been under investigation. We found, in our current study, that the peritumoral injection of amifostine is well-tolerated and effective in preventing radiation-induced proctitis. In addition to intracavity peritumor injection of amifostine, we utilized off-axis applicator and recalculated the adjusted RBE and dose distribution curve of ^252^Cf radiation source, to deliver more precise ICBT. As in Phase III, all the novel theories and techniques applied in our clinics, safety of ICBT in our institute has been significantly improved, which is highlighted by that in patients with T2 tumor G2 LAC occurrence has been significantly reduced, while there were no G3 LAC in patients with T2 and T3 tumor.

In Phases II and III, the initial response to conventional CRT is the key to decide the ICBT dosing because it is closely related to local recurrence rate and prognosis after definitive therapy. One might wonder why tumor regression evaluation in this study was performed at 2–3 weeks post CRT while standard-of-care tumor regression evaluation was performed at 4–6 weeks post CRT. As EBRT utilized in current study was in neoadjuvant setting, we considered that ICBT is necessary for all patients included in this study, regardless of the initial response. Even for the patients who could presumably achieve CR in the neoadjuvant setting, ICBT was still a powerful local treatment when there was no surgical removal of original lesions. We considered EBRT and ICBT as a continuous and definitive radiotherapeutic regime, other than two phases of a whole therapeutic strategy, such as conventional neoadjuvant chemoradiotherapy followed by surgery. Historically, ICBT regime shifted from an add-on therapy to EBRT to a fused therapy within the EBRT in treating cervical cancer, suggesting the shorter interval between EBRT and ICBT is beneficial for local control and survival. In the previous clinical observation and Phase I of this study, we found that the tumor regression at 2–3 weeks after EBRT was highly correlated with clinical response at 4–6 weeks, and clearly indicated the responsiveness to conventional radiotherapy. Therefore, in Phases II and III of this study, we evaluated initial response earlier at 2–3 weeks after completion of EBRT.

Considering the good prognosis of those rectal cancer patients who achieved cCR after neoadjuvant CRT, the watch-and-wait surveillance strategy has been investigated recently and can be considered as a good comparison for our study (12). In a prospective non-randomized controlled trial adopting a watch-and-wait strategy, only 1 (5%) of 25 patients developed LR. Two-year cumulative DFS and OS were 89 and 100%, respectively, which was not statistically different from those of the control cohort (13). Other trials suggested a high rate of LR (28 of 90, 31%), with 26 patients (93%) proceeding to salvage therapy. However, there was no difference in systemic relapse rates between patients with or without LR (13 *vs.* 18%), and 3-year DFS and OS were 78 and 88%, respectively (14). A systematic review and meta-analysis that identified 23 studies including 867 patients also showed that there was no significant difference between patients managed with watch-and-wait and patients with clinical complete response treated with surgery in terms of non-regrowth recurrence (RR 0·58, 95% CI 0·18–1·90), cancer-specific mortality (RR 0·58, 95% CI 0·06–5·84), disease-free survival (HR 0·56, 95% CI 0·20–1·60), or overall survival (HR 3·91, 95% CI 0·57–26·72) (15). For those who achieved over 80% tumor regression in our study, the 4-year DFS and OS were 78 and 88%, which is comparable to patients who received watch-and-wait or stand-of-care treatment in the previous study, suggesting that individualized ICBT is as effective and safe as other definite therapeutic strategies.

Our current study also includes patients with intermediate initial response to CRT, which was defined as tumor regression between 50 and 80%. This population of rectal cancer patients usually have intermediate prognosis due to the relatively lower sensitivity to radiotherapy. Thus, clinical oncologists and radio oncologists set to investigate how to improve treatment efficacy of LARC patients by incorporating novel brachytherapy techniques. Previous reports showed promising local control and survival in LARC patients adopting contact X-ray brachytherapy or high-dose rate endorectal brachytherapy (HDREBT) using ^60^Co or ^192^Ir (5, 16, 17). Noteworthy, these techniques, including contact X-ray radiotherapy or HDR Ir-192 isotope brachytherapy (used off-axis multichannels applicator), were usually allowed only if the tumor has regressed above 80%. If tumor has regressed less than 80%, immediate salvage surgery should be recommended to the patients (18). The results from our current study even have comparable local recurrence and survival to rates reported for standard treatment with neoadjuvant CRT in combination with surgical resection (19–21). Moreover, in terms of functional outcomes, all patients treated non-operatively had sphincter preservation, whereas usually almost half of patients in the standard care required a permanent colostomy. In contrast, our current study showed better rates of 4-year PFS and OS in digits, regardless of the initial response rate, even without any radical surgery procedures. Our results indicate that for patients without surgery opportunity or willing, individualized ICBT regime is a plausible option.

As for rectal interstitial implant, it was used to solve the dose deficiency of deep rectal tissue with ^192^Ir γ-ray for early stage T2 patients and patients with residue tumor after contact X ray/EBRT. Before 2009, we applied rectal interstitial implant using ^192^Ir γ-ray for patients with residue tumors after ^252^Cf neutron ICBT/EBRT. However, local control was achieved in less patients (less than 50%). After 2009, we applied rectal interstitial implant using small size ^252^Cf neutron source for patients with residue tumors. Noteworthy, local control was achieved in more patients (14/22,63.7%).

Given a number of previous studies have correlated complete pathologic response with lower recurrence rates, as well as improved DFS and OS, patients who have less than 50% tumor regression after CRT are usually considered to be associated with poor prognosis, suggesting this population is the key target in need of more powerful treatment to overcome therapeutic resistance and improve survival (3). Hence, to further improve the therapeutic efficacy of those population, study is undergoing in our facility to give an innovative ^252^Cf neutron rectal interstitial implant after boric acid solution intratumoral injection technology to increase the local radiation dosage by creating boron neutron capture effect. Our pilot study showed that this novel technique can further improve the treatment efficacy of T3 tumor or radiotherapy-insensitive tumor (initial response <50%).

Another limitation of current study is the lack of standard-of-care control group, which ideally should be surgery. The obvious reason is we can only test this therapeutic strategy in a cohort of patients who are inoperable or refuse surgery in this proof-of-concept study. As we have shown very promising outcomes of current individualized ICBT protocol in treating low-lying rectal cancer patients, future prospective controlled study is granted.

## Conclusion

Concurrent chemo-EBRT combined with individual ^252^Cf neutron ICBT protected by IPIA showed promising efficacy and safety in treating low-lying T2 and T3 rectal adenocarcinoma patients. Furthermore, it also extended the indication of curative concurrent chemoradiotherapy for T2 and T3 low-lying rectal adenocarcinoma greatly compared with other non-surgical radiotherapy methods.

## Data Availability Statement

The original contributions presented in the study are included in the article/supplementary material. Further inquiries can be directed to the corresponding authors.

## Ethics Statement

The studies involving human participants were reviewed and approved by the ethical review board of Daping Hospital, Army Medical University. The patients/participants provided their written informed consent to participate in this study. Written informed consent was obtained from the individual(s) for the publication of any potentially identifiable images or data included in this article.

## Author Contributions

XL and MXL conceived and coordinated the study. XL, MXL, and YX wrote and revised the paper. LS, JL, and QZ performed Californium-252 Neutron Intracavitary Brachytherapy to patients. CL followed up patients and analyzed the experiments. MJL, LZ, and XD followed up patients. All authors reviewed the results. All authors contributed to the article and approved the submitted version.

## Funding

National Natural Science Foundation of China (Granted Number: 81902671).

## Conflict of Interest

The authors declare that the research was conducted in the absence of any commercial or financial relationships that could be construed as a potential conflict of interest.

## Publisher’s Note

All claims expressed in this article are solely those of the authors and do not necessarily represent those of their affiliated organizations, or those of the publisher, the editors and the reviewers. Any product that may be evaluated in this article, or claim that may be made by its manufacturer, is not guaranteed or endorsed by the publisher.
